# Perceptions of mental workload in Dutch university employees of different ages: a focus group study

**DOI:** 10.1186/1756-0500-6-102

**Published:** 2013-03-18

**Authors:** Judith T Bos, Nathalie CGM Donders, Koos van der Velden, Joost WJ van der Gulden

**Affiliations:** 1 Department of Primary and Community Care, Centre for Family Medicine, Geriatric care and Public Health, Radboud University Nijmegen Medical Centre, PO Box 9101, 6500, HB Nijmegen, the Netherlands

**Keywords:** Age differences, University employees, Academic employees, Mental workload, Stress, Greedy organisation, Focus group interview

## Abstract

**Background:**

As academic workload seems to be increasing, many studies examined factors that contribute to the mental workload of academics. Age-related differences in work motives and intellectual ability may lead to differences in experienced workload and in the way employees experience work features. This study aims to obtain a better understanding of age differences in sources of mental workload. 33 academics from one faculty discussed causes of workload during focus group interviews, stratified by age.

**Findings:**

Among our participants, the influence of ageing seems most evident in employees’ actions and reactions, while the causes of workload mentioned seemed largely similar. These individual reactions to workload may also be driven by differences in tenure. Most positively assessed work characteristics were: interaction with colleagues and students and autonomy. Aspects most often indicated as increasing the workload, were organisational aspects as obstacles for ‘getting the best out of people’ and the feeling that overtime seems unavoidable. Many employees indicated to feel stretched between the ‘greediness’ of the organisation and their own high working standards, and many fear to be assigned even less time for research if they do not meet the rigorous output criteria. Moreover, despite great efforts on their part, promotion opportunities seem limited. A more pronounced role for the supervisor seems appreciated by employees of all ages, although the specific interpretation varied between individuals and career stages.

**Conclusions:**

To preserve good working conditions and quality of work, it seems important to scrutinize the output requirements and tenure-based needs for employee supervision.

## Findings

### Background

Almost one third of the European employees state that their work affects their health. Stress was identified as one of the main causes [1]. Work-related stress is generally considered to be the product of an imbalance between environmental demands and individual capabilities, and depends on the person’s appraisal of the work environment [2] and the anticipated ability to cope with the problems that cause stress. The term often used to describe this situation is ‘workload’. Researchers identified several dimensions in the construct ‘workload’, including mental and physical components, and quantitative and qualitative components [3]. Quantitative workload is concerned with the amount of work someone has, while qualitative workload has to do with the difficulty of the work. In university settings among scientists, a mixture of qualitative and quantitative mental workload can be found. Multiple roles are expected of faculty, including excellence in classroom teaching, accessibility to individual students outside of class, productivity in traditional research or other creative endeavors, acquisition of funding, et cetera [4].

Socioeconomic and political developments have increased the mental workload among university employees in the last few decades [5-8]. Public services, including universities, are increasingly being redefined as market commodities, a means of serving society. This introduced many organisational and technical management changes [9] among which increased competition between universities for students and research income. To improve efficiency and to achieve ‘value for money’, universities across Europe are increasingly interested in strengthening the accountability and responsiveness of higher education to society [10,11]. This resulted in the contemporary organisation of universities, characterized by a “post-Fordist” job design: greater flexibility—achieved through the use of new technologies which offer the possibility to work at home just as easy as in the office—, higher levels of autonomy, more responsibility for employees to get the job done, and a shift from attention for the number of hours worked to targets and deadlines that have to be met [12]. Although autonomy and flexibility generally are beneficial, for instance for combining the demands from the work and home, post-Fordist organisations (including universities) are also recognized to be ‘time-greedy’ [4,12]. Workers are charged with personal (or team based) deadlines, which stimulates the tendency to working overtime [12]. The workload of university employees indeed considerably exceeds a typical workweek, resulting in universities having turned into “greedy institutions” [4]. Coser [13] introduced this term to describe institutions “that are not content with claiming a segment of the energy of individuals but demand their total allegiance” (p.198).

Research shows that 17 to 55 percent of the employees in European universities experience problems with workload [14-16]. There is evidence of a negative association between prolonged high workload and mental and physical health [17,18] and work motivation [19]. Moreover, many university employees report working overtime at home [20], which may lead to problems such as work-home conflict [21,22] and an increased turnover intention [23]. These findings emphasise the need to reduce workload to an acceptable level.

Many studies have been conducted into the factors affecting workload among university employees, which have revealed various potential bottlenecks. Two major groups can be classified: (i) characteristics of the organisational climate and educational policy, such as rising student numbers and administrative duties, and (ii) characteristics that are intrinsic to the job, like frequent interruptions and compromised personal priorities [15,22]. Several studies distinguished between functional levels, like assistants and other academic staff versus participants with a professorship or academics versus general staff [6,8]. However, so far, little attention has been given to age differences.

Work motivation and work goals and possibly also work behaviour seem to be associated with calendar age [24]. In general, younger employees seem to focus on career advancement, salary and recognition, while older employees prefer the use of own skills (self-actualisation), to help other people and to contribute to society [25,26]. Although differences in age do not seem to cause differences in performance, age-related declines in fluid intellectual abilities (e.g. working memory, abstract reasoning) may impact on experienced mental workload [24]. Age-related differences thus may affect the way employees experience their work, the work features, and work outcomes like work stress. Uncovering these views will help to optimise a healthy and stimulating working environment for university employees of all ages.

### Research context

Universities across Europe have adopted organisational strategies for planning and control (e.g. more emphasis on output indicators) that are commonly found in the private sector in order to meet socioeconomic and political developments [10,27]. In 2007 the Dutch government radically changed university funding. Since then a large part of the funds that the government previously awarded directly to universities has been made available through funding agencies. Scientists have therefore had to invest more effort in acquiring research funding. In addition to this, major organisational changes have been implemented in European universities in recent years to increase internationalisation. Examples are the introduction of a bachelor-master structure, teaching in English [28] and the European Credit Transfer System (ECTS); the credit system that facilitates mobility and academic recognition between universities within and between countries [29].

### Aims

This study was conducted to investigate age differences in sources of (qualitative and quantitative) mental workload among scientists. We explored: (1) aspects of work that are experienced most positive and (2) factors that were perceived to lead to an excessive mental workload. Measures of workload may use an objective or perceived operationalisation [3]. Objective measures are concerned with the verifiable amount or difficulty of the work, while perceptual measures focus on the amount of work relative to individual stamina and energy, capabilities and available time Because high objective workload is not necessarily something to address in interventions, we focussed on perceived workload. The focus group methodology was chosen to collect data, because it is ideally suited for uncovering perceptions and underlying ideas [30]. Moreover, interactions between the participants will aid exploring the occurrence of high workload in greater detail [31]. Qualitative content analysis revealed a set of themes which will be reported sequentially. The composition of the focus groups was based on calendar age to gain an understanding of the age-related differences. The two research aims will be addressed successively, emphasising the causes of workload as those were most extensively discussed during the interviews.

## Methods

### Participants

The investigation was entrusted to the University’s Occupational Health Service. In early 2009, the management of the faculty granted permission for the study and invited all scientific personnel (N=270) by means of an e-mail containing detailed information about the purpose and design of the study. Anonymity was guaranteed. Within three weeks, 40 employees decided to participate by sending an e-mail to the occupational physician (JG) who lead the focus group discussions. None of the participants had been previously seen by this occupational physician. Eight focus groups were conducted; each focus group comprised a limited number of participants (range 3–6 participants; mean 4) to allow sufficient time for each participant to express themselves fully. Six employees were not able to participate after all due to personal, non-work related reasons. One employee did not participate for unknown reasons. Table 1 presents an overview of the age, position and sex of the participants. More women (22) than men (11) attended the focus group interviews, but men and women were proportionally spread over the focus groups.

**Table 1:** Participants

**Age group**	**FG number**	**Age**	**Title**	**Sex**
<36 year	4	26	PhD candidate	Female
		27	PhD candidate	Female
		29	PhD candidate	Male
		29	Assistant professor	Male
		30	Assistant professor	Female
		32	Assistant professor	Female
	5	27	Teacher	Male
		28	Teacher	Female
		32	Assistant professor	Female
		33	Assistant professor	Female
		35	Assistant professor	Female
36-44 year	3	36	Associate professor	Male
		38	Assistant professor	Female
		40	Teacher	Female
		41	Researcher	Male
	6	36	Associate professor	Male
		38	Assistant professor	Female
		42	Assistant professor	Female
	8	37	Assistant professor	Female
		40	Assistant professor	Female
		40	Researcher/ Assistant professor	Female
45-54 year	1	44	Researcher	Female*
		46	Assistant professor	Male
		49	Associate professor	Female
		50	Assistant professor	Female
		52	Researcher	Male
	7	46	Assistant professor	Male
		52	Assistant professor / coordinator internship	Female
		58	Assistant professor	Female*
≥55 year	2	56	Professor	Male
		58	Assistant professor	Male
		59	Professor	Female
		59	Administrator	Female

* Due to calendar difficulties, these participants were included in another age group.

### Procedure

Focus groups should have a homogenous composition, while leaving enough heterogeneity to allow for contrasting opinions [30]. Homogeneity was achieved since all participants were scientific personnel at a faculty of a Dutch university and willing to discuss causes of work overload and its possible solutions. In addition, they were divided into four age groups (<36 years, 36–44 years, 45–54 years and 55 years and older). Heterogeneity was strived for by explicitly inviting people with and without feelings of work overload as well as by including employees from different departments with different positions and responsibilities in each of the focus groups.

The focus group interviews started with a brief introduction, presenting the aim of the study, how the information would be used and by asking permission for audio recording the interview. Consent was obtained from all participants and everyone agreed to the request to keep the discussion confidential. Participants were encouraged to discuss, rather than to find consensus, and to deeply explore underlying reasons for mental workload. The interviews lasted 1.5 to 2 hours. At some point during the focus group interviews, participants were asked to write down the 2 most positive aspects of their work.

An occupational physician (JG) acted as moderator and guided the discussion in the sense of keeping it on topic and a researcher (JB) observed and took notes. During the interviews the emphasis was on aspects that increase the workload. Three questions were presented to the participants: 1) which work-related characteristics contribute to your workload? 2) which non-work-related characteristics contribute to your workload? and 3) what personal strategies do you employ to manage workload?

The topics to be discussed were selected in advance based on literature findings on determinants of workload among university employees [6,32,33]. The wording of the questions and the order in which the topics were discussed, were adapted to the responses of the participants. Moreover, the content of the interviews was adapted to information gained in previous interviews. After each focus group interview an extensive interview report (by JB) was presented to all participants for approval to assert ‘member checking’ [34]. In addition, all tape recorded interviews were written verbatim by a research assistant to extract quotes to illustrate findings. In the Netherlands, ethical approval for an interview or survey study with employees is not required (see for Dutch legislation http://www.ccmo-online.nl/main.asp?pid=1&taal=1).

### Data analyses

The individual lists with two most positive aspects of work were analysed and compared to reveal the five most positive aspects of work in each of the age groups.

All interview reports were analysed using the qualitative software program ATLAS-ti 6.1.4. Verbatim transcripts were analysed according to qualitative content analysis [35]. All files were independently coded by JB and the research assistant. Afterwards, consensus was obtained by discussing the reasoning of the coding. During analyses, initial codes derived from literature on causes of workload among university employees, were supplemented with codes that arose from analysing the transcripts. Some codes appeared to be closely related and were combined during analysis. The final code list consisted of 30 codes. To ensure that the allocation of codes was identical in all reports, in a final round, all quotations were separately checked per code and then discussed by JB and the research assistant. Two researchers (JB and ND) independently organised the codes into categories based on their shared content, after which themes were identified by discussing the categories [36].

## Results

### Factors that are experienced most positive

Interaction with colleagues and students and autonomy (in time or work) were mentioned as being pleasurable by most age groups (Table 2). Most participants perceived teaching as a wonderful and creative profession and they liked disseminating knowledge to students and discussing the subject with them. In addition, almost all age groups valued having “interesting work”: they considered their job challenging (35–44), liked the intellectual environment (45–54) and enjoyed being pioneering in their field (≥55). Moreover, for the two youngest age groups the possibility to continue learning was considered a positive aspect of work, while the two oldest age groups highly appreciated the content of the work, i.e. their disciplines and the related research and teaching tasks. Due to the limited number of participants aged ≥55, for this age group only aspects that were mentioned twice or more, are presented.

**Table 2:** The five most positive aspects of work according to employees from four different age groups

**<36 years**	**36-44 years**
1. Autonomy (work)	1. Colleagues
2. To continue learning	2. Interaction with students
3. Colleagues	3. Interesting work
4. Autonomy (time)	4. Autonomy (work)
5. Interaction with students/ teaching	5. To continue learning
**45-54 years**	**≥55 years**
1. Colleagues	1. The content of the work
2. The content of the work	2. Challenging work in an academic atmosphere/ be innovative or pioneering
3. Interaction with students	3. Teaching
4. Intellectual environment / use creativity	4. Colleagues
5. Autonomy (work and time)	

### Factors that are perceived as increasing the workload

Three themes and six categories were distinguished among the reported factors influencing perceived workload (Table 3) Because the aim of this study was to detect differences between age groups, the results, presented for each theme, are described from the observed differences. Citations are marked with sex (f/m), focus group number (FG1-8) and age group (<36, 36–44, 45–55 and ≥55).

**Table 3:** Themes and categories resulting from qualitative content analysis of the verbatim transcripts, in order to reveal factors that were perceived as leading to an excessive mental workload (research aim 2)

**Theme**	**Category**	**Codes**
Organisation	Organisational aspects as obstacles for ‘getting the best out of people’	Coaching and supervision
		Challenges in the work
		Faculty communication
		Embedding of feedback
		Pressure linked to publications and grant applications
		Strict rules
		Autonomy*
	Greediness of the organisation	Department atmosphere
		A feeling that the organisation does not care about its staff
		Work-family/ life balance
		Work and life experience
		Promotion prospects
Aspects of work	Overtime seems unavoidable	Organisation of education
		Student interaction
		Organisation of the work
		Inadequate resources
		Faculty policy
		Unique knowledge
		Administrative/ organisational tasks
		Too much work to do
		Frequent interruptions at work
		Working at an university*
	Barriers to built a career	Working in separate projects
		Finding time for research*
		Working relationships*
Personal approach	Personality	Work engagement
		Overcommitment
	Personal adaptation	Long working hours
		Personal organisation of the work
		Personal needs

* These codes were not further elaborated because neither age differences nor unique results were present.

#### Theme 1 – organisation

This theme concerns both personal support from direct supervisors and organisational management tools.

**Organisational aspects as obstacles for ‘getting the best out of people’** Participants indicated to lack coaching and supervision from the university in their goal to provide good teaching and research. Being highly motivated, this generated feelings of stress and frustration. Participants mentioned the impossibility to fully benefit from each other’s knowledge and skills. Time constraints seem to hinder discussion or even having lunch together, partly because colleagues have different teaching schedules. Those <36 and 36–44 lack the possibility for a joint reflection on ‘science’.

I was very much confronted by that. That one person who you could still learn something from or with who you could still achieve something together was too busy. (…) And exploring new pathways together happens less than I had hoped and had thought. [f1-FG4:<36]

All participants considered substantive improvement in coaching and supervision possible. For example, the ≥55 sometimes feel abandoned: they lack real solutions for the problems they face. They also mentioned the insufficient managerial opportunities for them as supervisors to protect their co-workers from becoming overloaded. Participants aged 36–44 and 45–54 feel their supervisors often have insufficient knowledge of the subject they are studying, although some report to benefit from their supervisors skills in steering the process and their helicopter view. In response to the question “what exactly is good leadership”, it was answered:

Really very simple things, that someone just listens to you, that somebody asks about your experiences and so not just … Also invites you to say something about your experiences, enquires about your ambitions and goals, has an eye for your future. (…) You simply behave differently if you notice that the contact is good. You grow as well. [f1-FG8:36–44]

Moreover, perhaps indirectly important for the perceived workload, the 45–54 argued that feedback regarding performance should also include giving compliments, which is not time consuming but very motivational.

I said: if you get good education evaluations, and I always get those, then you never hear anything about that. (…) So I said: ‘I really don’t like that. I think that you could sometimes simply … I want a pat on the head. Once in a while: “Hey, you’re doing that really well”. If you genuinely get good evaluations, then I don’t want that to go unnoticed’. [f1-FG1:45–54]

A lack of social cohesion might further complicate the possibility to motivate and to challenge each other and to learn from each other.

I myself compare that very strongly with [another institute] that is even far more pushy than any other university in the Netherlands. (…) But I have the idea that a lot of energy is bubbling there. (…) There is far more critical mass within each subarea. That is often directed from above but there is also a lot of coercion. All of the staff attends the weekly reading group or all junior researchers must make sure that they make a brief report twice per month. Those sorts of things. [m3-FG6:36–44]

Another issue mentioned was that information, relevant for optimal performance, is sometimes insufficiently communicated within the faculty. Younger participants noticed that information on obtaining their teaching certificates or on grading the students’ exams was missing, leading to underperformance and difficulties with career planning. Older participants indicated to lack information on compensation for additional tasks, on obtaining grants or on realising policy. Although some employees liked having the opportunity to think along about policy issues and to contribute to the better functioning of the department, this was sometimes considered impossible due to the perceived lack of information.

I do not have the knowledge for it and I have insufficient information about what happens in the faculty and in the department. (…) The consequence of this is that we cannot assess the management process well and cannot creatively think with them. [m3-FG6;36–44]

Several participants lacked a clear embedding of feedback and coaching. The <36 and 36–44 indicated that rules regarding the introduction and training of new or young colleagues are missing, while employees aged 45–54 mentioned that the coaching of young or new colleagues often has to be given on a voluntary basis.

In addition, two aspects derived from national policy were indicated to hamper pursuing a career: the research performance system and the pressure regarding writing research proposals to apply for grants. The research performance system is perceived by most of the participants as an ‘inflexible punishment system’, with too strict rules, not contributing to pursuing a career, and increasing the work pressure.

The faculty board told me “now sir, we believe that your research output is too low, we’re going to reduce your budget by 10%” and I experienced that as extremely frustrating and painful. Because I worked my butt off, up to and over the boundaries as it were, so you are left with the feeling that you really have tried your best but are nevertheless punished. [m1-FG2:≥55]

Writing grant applications was especially indicated to be a heavy burden because of the low probability of acceptance. The usefulness of such a system in terms of directing thoughts and elaborating ideas is recognised, but only by some younger workers (<36 and 36–44). The ≥55 emphasized that academic freedom has been restricted by this system of allocating resources. This not only forced them to write proposals in accordance with the wishes of fund providers, it also deprived them the opportunity to maintain job satisfaction.

Yes, well what I like about my work, um, is the level of challenge in this area. Um, the variety both in terms of research and education and the freedom that you have in, let’s say, the moment that the variety comes under pressure and then threatens to become a drag. The freedom you have then to adjust the content of your work such that you once again have a feeling of satisfaction. So that is what people, I think, normally mean by ‘academic freedom’, although of course in recent years, decades that has been significantly curtailed. [m1-FG2:≥55]

**The greediness of the organisation** Employees feel that the faculty board is counting on their responsibility and loyalty too much. For instance, employees of all ages indicated that there is an atmosphere at the department in which it is considered as normal to structurally work overtime by both the management and many colleagues. This causes feelings of not being valued and taken seriously among all ages. Participants told that it evoked the feeling that the organisation does not care about them, even when they largely exceed the requirements.

What concerns me somewhat is that I have the idea the prevailing attitude is that it is normal to invest your private time in work, that you are constantly seeking the boundary and that you are doing research in the evening, so to speak. (…) Because it is considered normal that you are so engaged about your work. [f4-FG4:<36]

So what strikes me is that if somebody were to count up his holiday days, it would be impossible for him to use them all. Take me for example because this year I had 26 holiday days left. And they have simply disappeared, evaporated into thin air. Simply because my work did not permit me to take them. And that does not only apply to me. [f2-FG2:≥55]

Another point brought forward is ‘career progression’. Some of the young participants indicated to experience conflicts between building a career and starting a family and they find it difficult to find a proper work-family balance. Older workers noticed this problem as well.

I hear a lot of stories: “people who are on the point of splitting up” and “young women who dare not start having children because their work does not allow for that” and I feel responsible for these young people. [f4-FG2:≥55]

Participants indicated that time investment and working abroad is beneficial for career progression, but that the family and life situation strongly determines how these requirements can be dealt with. Young participants, even without children, perceive that their (older) supervisors really push them to invest in their career. When families have been formed and children are born, it becomes more difficult to work at home or to work longer hours when necessary. This sometimes evokes feelings of frustration, although employees of all ages recognized that the experience of having children can help to break free from work.

Participants also stated that managerial decisions are often imposed top-down, without appropriately taking into account the impact on employees. The responses seem to differ between age groups: while many of the younger workers experience considerable stress because they feel they have to prove themselves to ensure their promotion prospects, some of the older employees are reluctant to follow all directives from the board, knowing that they will not be fired easily.

Every 3–4 months I have to hand in a progress report. (…) But then you want to do it well and so you spend time on it again (…). These are things that detract too much from the core task. The scientific staff (…) often say ‘you need to take that administration with a pinch of salt sometimes’. But the administration is the one that deals with the extension of your contract and the raise of your salary scale. [m2-FG4:<36]

Despite great efforts on the part of employees, promotion prospects are perceived as very limited except in the oldest age group: postdoctoral appointments, permanent contracts and the position of associate professor are all restrictedly awarded.

Now you name that [a broader set of responsibilities] ‘career’ and ‘climbing’ but that does not necessarily have to be translated into you also getting a different status. [f3-FG8:36–45] <laugh> That we are all laughing so loudly is clearly a sign of that that is just …, in fact you could also be very angry about it. [f2-FG8:36–45]

#### Theme 2 - aspects of work

This theme involves the work to be done by the scientific staff of the university. To build a career within a university setting, it is important to conquer a place in the scientific community by teaching, publishing and presenting on research within a specific domain. However, aspects of working at the university make it nearly impossible to avoid regularly working overtime and hinder building a career.

**Overtime seems unavoidable** In general, participants from all age groups appointed similar work aspects that increase the workload to uncomfortable levels. The oldest workers noticed a substantial change in the organisation of education over time. The courses are very compressed now in order to meet new requirements concerning the ECTS, and they regret that much less time is available for good and meaningful interaction with students.

I still think back with sadness, although that was also not good, that was too casual. [m1-FG2:≥55] (…) And I fully realise, it was a blissful situation. That can also no longer be sold to the outside world. [m4-FG2:≥55] But for the students that is a shame. Because personally I think it was very good like that. [f2-FG2:≥55] It was also character forming. [f3-FG2:≥55] Education, I am always educating. [f2-FG2:≥55]

Other changes in the organisation of the work seemed to limit resources and increase workload too: i.e. the faculty policy regarding the number of teaching hours (increased) and the number of preparation hours assigned (decreased). Moreover, the number of students increased and, according to many of the participants, the intellectual capacity of the first year students over the past 10–15 years decreased. Reviewing exams is experienced as an increasingly more comprehensive job, especially since the faculty board has decided to additionally offer students partial exams as a tool for developing a more effective study method. Participants indicated that this increase in workload cannot easily be avoided. Courses cannot simply be passed on to someone else because unique knowledge is required. In addition, administrative and organisational tasks impose considerable demands on the time available. The youngest workers feel they sometimes have to write superfluous progress reports, while the participants aged 36 and over indicated that their time is often fragmented due to secondary tasks and meetings. As a consequence, a sense of a continuous time deficit causes feelings of working below potential and hence of frustration.

What I also find really annoying is that there is not the room to deliver the quality you think that is possible to deliver. (…) And that, as you say, there is also scarcely the room to orientate yourself more broadly and to get to know people. (…) The basic responsibilities here tie you down so much that there is little opportunity to get out and about. [f3-FG8:36–45]

Participants mentioned to regularly work at home, which reduces the distinction between leisure and work time. The reasons for this are varied. Most mentioned is that it is a necessity to work during evenings and weekends; they have too much work to do, this is the only way to get the job done. This does not seem to be a definitive solution, as apparent from the observations of 36–44 year olds: they had to stop working overnight, because it exhausts them too much. Another common reason is the turbulent environment. As qualitative workload is high among university faculty, concentration is necessary to do the job well. However, concentration is frequently interrupted by students coming in to ask questions, telephone calls and e-mails to be processed. Many people share a room with colleagues who consequently disturb each other.

I am in an office with two other lecturers. And it is really difficult to simply work quietly, I cannot do it. If I am alone then I can but then one or the other lecturer can come in at any moment. And he comes in with a lecturer, or with two or with a group. Then I can just as well go home, then I still can’t do anything between the lectures. And in turn, I disturb the others as well. [f2-FG7:45–54]

**Barriers to build a career** To conquer a place in the scientific community, academics need to publish and present within their specific domain. However, money for research is always linked to separate projects and time should in principle be used for that particular study, which should be finished within set time limits. Some participants aged 45–54 stated that this implies that it is hard to find time for additional requests that sometimes arise from earlier projects (writing a book chapter, giving a presentation etc.). These should be conducted in one’s own time or be refused. Refusing such requests is considered undesirable: it is an essential part of acquiring a place within the scientific community and a matter of professional pride.

#### Theme 3 - personal approach

This third theme covered individual changeable and unchangeable opportunities to deal with workload. The personal characteristics mentioned were related to personality or the personal adaptation to high mental workload.

**Personality** The tendency to be highly engaged to work and perform very well is ubiquitous among our respondents.

But all of us also work for an eight or a nine [roughly comparable to A-grade]. We want to deliver work that is as good as possible. That is also why we were given this job. And perhaps we ought to learn that for some things a six or a seven [roughly comparable to B-grade] is also enough. [f2-FG2:≥55]

The focus on getting sufficient job satisfaction seems to be one of the causes of high workload through the great effort that people show.

If you do not work in the same manner as we do, namely always starting up new projects, making changes to the material you lecture on, then the work is not challenging enough. [f2-FG3:36–44]

The feeling of high workload is futher enlarged by the high work standards and overcommitment many: participants feel that the preparation of teaching seems never finished and that it always can and should be better. Some indicated that teaching, compared to doing research, exhausts them.

It is also part and parcel of the subject we teach. You have never finished, it can always be better. And what you have in your head is always better than the final result. We have a creative discipline and that is also what makes it so attractive. [f1-FG3:36–44]

**Personal adaptation** This (over)commitment and work engagement often results in -what appears to be experienced as- ‘voluntary overtime’ for many participants, resulting in working long hours. Overtime caused by the personal way of organising the work seems to be perceived as less burdensome than involuntary overtime (e.g. due to orders from the management).

Yes I do exceed it and I have the idea that I choose to do that. Because I could do it within my work time of 38 to 40 hours per week but I just choose to invest more time in things and then I am also working on it in the weekend. [m2-FG4:<36]

But for others it is simply a necessity to finish the work in time.

For me it is logical to grade essays during my holiday. How else could I have succeeded? [f1-FG3:36–44]

Other attempts to cope with the imbalance between job requirements and (personal) work goals are: refusing full-time contracts and to have the courage to occasionally refuse tasks imposed by the management. In addition, while many employees younger than 54 years indicated to need some time to recover *after* a day of work, some ≥55 indicated they occasionally needed time to recover *during* the working day.

If I have given a long lecture then I simply need a breather. Then you simply cannot do anything else for a while. Phew. [f2-FG2:≥55]

## Discussion

This study aimed to gain more insight into the factors that affect mental workload among university faculty staff and the differences between employees of various ages with regard to this. Focus group composition was therefore based on age. Employees of all age groups indicated that they highly appreciated the qualitative workload (i.e. having challenging work, working in an intellectual environment and pioneering in the field), but that they perceived the quantitative workload as a major burden: they were highly satisfied with their work tasks but not with the amount of work and the organisation of the work. Houston et al. [7] argued that academics might be attracted to university careers despite high workloads, because they value flexibility, autonomy and the appreciation of contact with students and colleagues outside their university. The answer to our first research question (what factors make work enjoyable) seems to support this assumption, as participants in all age groups reported highly valuing work aspects like interaction with students and colleagues and autonomy.

Among the causes of high workload, three broad themes were distinguished: aspects of the organisation, aspects of work, and personal. These findings are discussed in the next section.

### Factors that are perceived as increasing the mental workload

Participants of all age groups seemed mainly burdened by aspects of “organisation”: lack of support, feedback and supervision, and discontent with the current research performance system and the distribution of research funds. Participants generally perceived many policies as obstacles for good performance and career progression, which both were seen as causes of a high workload in other research as well [5,6]. In order not to squander talent, organisations should offer appropriate conditions [37], which in the case of university employees may consist of help in steering efforts or offering time management. Another option may be training on job crafting which has been defined as self-initiated change behaviours that employees engage in with the aim to align their jobs with their own preferences, motives, and passions [38]. In our study population, the influence of ageing seems most evident in employees’ actions and reactions, while the causes of workload mentioned seemed largely similar. Differences in reactions may at least be partly driven by tenure. Generally, younger employees felt obliged to respond to all kinds of requests in order to ensure career progress, while many older employees preferred to make an assessment of the requirements before complying with requests from the faculty board.

Participants mentioned “aspects of work” as causes leading to structurally working overtime and as barriers to build a career. Generally, employees of all ages experienced similar aspects of work that increased the workload to uncomfortable levels: too many administrative and organisational tasks, increased education-related workload, and a turbulent working environment were frequently mentioned. It was the imbalance between the variety of tasks assigned and the time awarded to accomplish them that causes most stress and frustration. According to Taris et al. [39], stress among university employees is induced by the combination of research and other tasks (teaching and management). They linked their finding to the idea that many academics primarily consider themselves to be researchers. Our research does not fully confirm this assumption. Most participants indicated that they considered their teaching tasks of great importance. However, many participants did feel frustrated. The preparation time for courses is often perceived as too short, and at least some of the time assigned to research has to be used to prepare the courses well, whereas the participants are assessed on their research output. Due to these circumstances, in line with Van Echtelt et al. [12] many participants feel that the use of overtime is unavoidable. Although working in the evening or weekends is experienced as requiring less effort and as less stressful than regular work hours [20] and may increase productivity (e.g. number of publications) [40], it can be a pitfall as well. It may give a sense of infinite time, making it tempting to postpone tasks and to take on additional tasks.

Participants developed a personal approach for dealing with the work demands. As long as the personal circumstances allow, most participants preferred to spend plenty of time on their work, often much more than required according to their contract. However, the older employees indicated that they need space for other personal interests than their work, although their family obligations have decreased. Our study revealed that participants who did not experience excessive workload often worked structurally too many hours. This “stress avoidance” strategy is also observed in other studies [7,15] and may express a personal characteristic that seemed common among the participants in all age groups: high commitment. Participants showed high intrinsic work performance standards and were very much involved with the students they serve. In addition, many participants indicated spending a lot of time on renewing and improving lectures and on commenting on essays in order to motivate and enthuse the students as well as themselves. Results from other studies among university employees also show a tendency towards perfectionism and setting high standards [7,15,41]. However, some studies showed that this apparently voluntary behaviour may be triggered by the way an institution is organised. By increasing both freedom and personal responsibility for final results, employees are provoked to work until the work is done [12,40]. Since some of the requirements for academic work is being “highly committed”, being eager to transfer knowledge that is most up-to-date, and being willing to explore the area of own interest in greatest detail in order to contribute to the expansion of knowledge, it is of great importance to provide supervision to academic staff of all ages and at all stages of the career.

### Consequences for practice and research

This study showed that although academics are quite positive about their jobs, there are many aspects that could improve their well-being at work in the sense of reduced workload, improved satisfaction and, according to some participants, even improved performance. Our participants consider it important to be treated well, e.g. by sufficient communication and support and an atmosphere of respect. Although employees with greater tenure and higher levels of education generally express less preference for leader structuring [42], we found that employees of all ages would appreciate improvement of support from their direct supervisor. Young participants desired more in-depth coaching and some aged 36–44 liked to be motivated and stimulated in their work by their supervisor, while many 45–54 year olds wanted coaching ‘on request’. Some of the oldest participants desired recommendations and tips on how to meet the numerous demands and at the same time more understanding from their supervisor for their need to engage not solely in university matters. So, employees of all ages prefer a supervisor that does not tell them what to do, but rather pay more attention to the development of work and career, including giving direction to the efforts on output and promoting the scientific debate within the faculty. Our results suggest that highly educated and highly motivated employees with seemingly advantaged positions and jobs, characterized by lots of variety, high autonomy levels and high workload, may benefit from some forms of guidance. It may also suggest that formally appointed supervisors or more experienced colleagues should be given the opportunity, both in time and by training skills, to provide the coaching and help asked for.

The contemporary requirements regarding accountability make it necessary for academic employees to repeatedly produce progress reports. Although such reports contribute to the insight into the performance of an organisation, they are time-consuming and thus leading to increased workload. It is recommended to critically evaluate the necessity, frequency and size of such reports.

Research has suggested that academics often feel satisfied with their jobs despite high workload as long as certain intrinsic needs are met (e.g. challenging and highly interesting work) [5]. This may explain why many participants indicate to work more hours than contractually agreed. Although academics indicate to work voluntarily in the evenings and weekends, it is questionable whether this is truly voluntarily: work simply has to be finished in time and promotion prospects depend on excellent performance (satisfied students, high quality research). Universities actually can only operate thanks to the high commitment of their employees. However, in order to preserve sufficient and well performing staff it is necessary to consider the demands from both education and research upon the employees: high workload and long hours are associated with diminished health [17,18], and the intention to leave [43], leading to drop out of employees.

As in the study of Houston et al. [7], participants tend to criticise ‘the faculty system’ for their high workloads. However, employees of all ages also have their own share in this: they appeared to have difficulty with or resistance to lowering their standards, because they expected reduced job satisfaction or because they did not know how to adjust their working methods. In fact, some participants continued to take on additional tasks, because they literally liked the work so much. Supervisors need to acknowledge the tendency among many subordinates to work very long hours, due to either self-imposed high devotion to work and/or by the desire to meet the often imprecisely defined tenure standards [40]. They should take greater account of the struggle within workers between well-performing according to own standards and limited time to reach these personal (and organisational) goals. In dialogue with the employee, supervisors may search for ways to keep the workload manageable. In addition, the employees need to adapt their working behaviour in order to obtain a better work-life balance. Such behavioural changes and recommendations from the supervisor probably will be different depending on age as the needs to adjust work-life balance differ for younger and older employees.

Recommendations for future research primarily include a repetition of this study in a few years, as then other results might be found. The ≥55 group now largely consists of people who chose to continue working. At present, the options for early retirement are limited. People, who in the past would have chosen to stop working because they wanted to quit, or because work demands became too high, now have to continue to work. However, as our study confirmed many earlier findings, future research should perhaps mainly be focussed on how academics of various age groups and/or various career stages prefer to be supervised and on the resources they need, including the resources that facilitate the proper performance of supervisors. Finally, the impact of (organisational) changes and (individual) interventions (e.g. attention to time management or job crafting) could be explored.

### Strength and limitations

The results are based on data obtained by qualitative research. Therefore, the results are not limited to averages. Individual patterns and outliers, as well as the reasons for these, are detected and may provide new insights relative to quantitative research. However, qualitative research is vulnerable for interpretation bias. Well-known strategies, described in the methods section, were used to reduce this disadvantage. Moreover, using the focus group methodology may also have prevented some individuals from participating, due to feelings of discomfort. People may expect to feel threatened by talking about their problems with others who are able to cope with the large amount of work, or who may be competitors in some way. Anticipated problems with the time investment needed may also have played a role.

For this study, in line with focus group methodology [31], quite homogeneous groups were composed using the method of homogeneous sampling [44]; scientists of one faculty were included, classified by age. This improved the possibility to capitalise on people’s shared experiences and facilitated the tracking of the causes of workload, but it limited generalizability. However, there is general agreement that the goal of qualitative research is *not* to generalize beyond a sample to the population [44]. The findings presented here can provide more insight into academic workload experiences at different ages and may serve as input for further research on factors associated with workload among university employees.

Gender seems to affect the way work is experienced [45] and the way in which workload is handled [46]. Demands outside the work, such as child care and household tasks, which reduce the possibility to recover from work, also appear to differ between the sexes [45]. As more women than men participated in our study, it is possible that the view of men is somewhat underexposed. Moreover, besides age, other age-related aspects, like tenure, career stage and family obligations may affect the way employees are able to cope with workload. Although classifying by age can conceal the influence of these aspects, we have chosen to investigate the influence of calendar age because of the recent interest in stimulating employees to work longer. Our findings suggest that in future research career stage may serve as an (additional) classifying factor. Moreover, attention to gender might be desirable.

## Conclusions

Contrary to expectations based on age differences in work behaviour and work motives, the calendar age of participants did not seem to have a major impact on the aspects that were considered workload-increasing. Calendar age did seem to affect the actions and reactions to the causes of workload mentioned, although tenure seems important too: e.g. young employees tend to consider their need to make a career when deciding whether or not to use opportunities to reduce the workload. Moreover, the tendency to perform (more than) well, combined with organisational characteristics that promote a performance culture, including output-oriented steering seemed to adversely affect the feelings of high workload.

Based on the results from this study, supervisors should bear in mind that a tendency to perfectionism may lead to exceptionally good results, but also to an excessive workload. Employees of all age groups indicated to appreciate more supervision, with method and content depending on work experience and position within the organisation. Moreover, it may be useful to consider the organisation and innovations of the study programme, including the working conditions of academics. The need for action seems justified because, in accordance with the literature, many participants felt unsatisfied with aspects of their work. This made them doubt about their ability to work for many more years or the desire among young participants to have such a job.

## Competing interests

The authors declare that they have no competing interests.

## Authors’ contributions

JTB participated in the design of the study, observed and took notes during the focus group interviews, performed the analyses and drafted the manuscript. NCGMD assisted with the analyses and commented on drafts of the manuscript. KV commented on drafts of the manuscript. JWJG designed the study, was the moderator during the focus group interviews and commented on drafts of the manuscript. All authors read and approved the final manuscript.
